# Recent Progress in Heterostructured Photocatalysts for Contaminant Removal and Solar Fuels Production

**DOI:** 10.3390/ma19214670

**Published:** 2026-11-02

**Authors:** Michalis K. Arfanis

**Affiliations:** Quality Control Department, Hellenic Pasteur Institute, 11521 Athens, Greece; m.arfanis@pasteur.gr; Tel.: +30-210-6478830

**Keywords:** photocatalysis, heterostructures, nanomaterials, nanocomposites, electronic properties

## Abstract

Advanced oxidation processes using photocatalytic materials are a very promising approach to overcome the shortcomings of anthropogenic pollution. Over the past decades, numerous studies have focused on titanium dioxide, zinc oxide, hematite, bismuth vanadate, and molybdenum disulfide, establishing TiO_2_ as one of the most effective photocatalysts, with low cost, low toxicity, and high chemical stability. The investigation of their combinations as binary composite materials showed that they can also be utilized in environmental applications. Still, the performance of all catalysts can be significantly improved if coupled with other nanomaterials, such as carbon nitride, carbon dots, covalent organic frameworks, transition metal phosphides, or MXenes. In this context, this review presents the majority of their binary combinations in heterostructured composites as an attractive strategy for enhancing the separation mechanism of photogenerated charge carriers and the production of reactive radicals. The various charge transfer mechanisms at the heterojunction of such materials are presented, while specific applications underline the improvement in their properties and their effectiveness in wastewater treatment, the neutralization of pathogens, hydrogen evolution, and the CO_2_ photoconversion to hydrocarbons.

## 1. Introduction

In recent years, widespread soil and water contamination, limited water resources, and the threats of climate change have led the scientific community to constantly explore new strategies for environmental remediation and green energy production. Among the different approaches, advanced oxidation processes using photocatalytic materials are suggested as effective methods to address the shortcomings of human activity. Depending on the nature of the contaminants, photocatalytic materials can either mineralize or transform them into valuable products through redox reactions. Titanium dioxide (TiO_2_, or titania) is generally accepted as one of the most powerful and efficient photocatalysts owing to its remarkable physicochemical properties and its performance across a variety of applications. However, the inability of titania to utilize the full spectrum of sunlight, as well as recombination of charge carriers at crystal defects, limits its integration into practical applications. Over time, various materials were proposed as alternatives, including zinc oxide (ZnO), iron oxides, bismuth vanadate (BiVO_4_), and molybdenum disulfide (MoS_2_). Notably, the preparation and examination of binary composites have also demonstrated their effectiveness in photocatalytic applications. In the last five years, numerous studies have appeared focusing on all possible combinations of the aforementioned catalysts with carbon nitride (g-C_3_N_4_), covalent organic frameworks (COFs), carbon quantum dots (CQDs), transition metal phosphides (TMPs), and MXenes for the preparation of highly efficient heterostructured photocatalysts (Table 1).

Thus, the scope of this review is to present all aspects regarding the fabrication, properties, and performance of novel and efficient heterostructured nanocomposite photocatalysts consisting of metal oxides, phosphides, MXenes, and carbon-based materials. First, the main optoelectronic properties of the pristine photocatalysts are described, with the aim of understanding both their advantages and limitations. Next, the different heterosystems of coupled semiconductors are examined, since the enhanced performance originates from the improved charge transfer mechanism at the heterojunction. Finally, the mechanisms of both contaminant degradation and solar fuels production are described, followed by indicative examples of specific state-of-the-art heterostructured composites, as marked in green in Table 1.

## 2. Photocatalytic Materials

**Figure 1:**
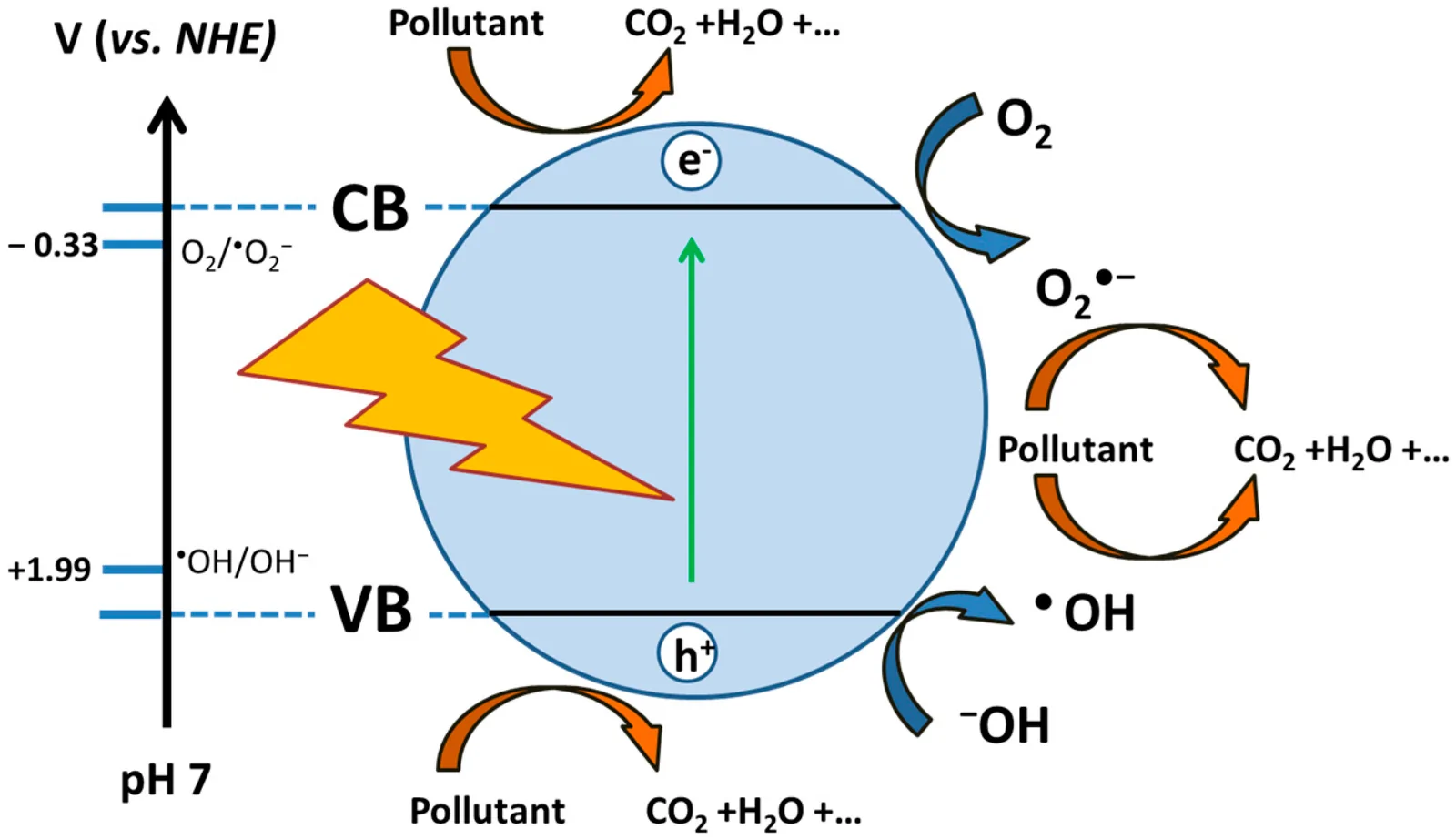
Photocatalytic mechanism for the formation of reactive radicals and the degradation of target molecules using semiconductors.

Heterogeneous photocatalytic reactions start when a semiconductor (SC) is irradiated with light of appropriate energy (hν), equal to its energy band gap (E_g_), as described in Equation (1) and illustrated in Figure 1. Instantly, a negatively charged electron (e^−^) is excited to the conduction band (CB), leaving a positively charged hole (h^+^) in the Valence Band (VB) [1]. If the VB and CB of the SC have suitable redox potentials, photogenerated h_ph_^+^ can react with hydroxyl ions (OH^−^) and e^−^ with molecular oxygen to generate reactive oxygen species (ROS), namely hydroxyl radicals (^●^OH) and superoxide radicals (^●^O_2_^−^), respectively (Equations (2) and (3)) [2]. In particular, the redox potentials of ^●^OH/OH^−^ and O_2_/^●^O_2_^−^ couples are 1.99 V and −0.33 V vs. *NHE*, respectively, at pH 7 [3,4]. These ROS can subsequently degrade organic molecules near the surface of the photocatalyst, ensuring progressive degradation over time, until complete mineralization (Equation (4)) [5]. Beyond the removal of persistent organic compounds in aqueous environments [6], ROS can also be utilized for the removal of NO_x_ and other volatile organic compounds [7,8], the inactivation of waterborne and airborne pathogens [9,10], or the production of solar fuels, such as H_2_ and hydrocarbons [11,12].

SC + hv → e^−^ + h^+^ (1)

h^+^ + OH^−^ → ^●^OH (2)

e^−^ + O_2_ → ^●^O_2_^−^ (3)

^●^OH + ^●^O_2_^−^ + organic compounds → … → CO_2_ + H_2_O + inorganic salts (4)

Since 1972, the photocatalytic performance of various inorganic semiconductors, with proper energy band gap and electrochemical properties, has been explored [13]. As mentioned earlier, titanium dioxide has been established as one of the most powerful photocatalysts for environmental applications. Specifically, titania is an n-type semiconductor with a band gap of approximately 3.2 eV, whose VB corresponds to an oxidation potential (V_ox_) of +2.53 V vs. *SHE* (for pH 7), and its CB to a reduction potential (V_red_) of −0.52 V vs. *SHE* (for pH 7), allowing the generation of hydroxyl and superoxide radicals [14,15,16]. Another appealing photocatalyst is the n-type semiconductor ZnO, which has an energy band gap equal to 3.37 eV, with V_ox_ ranging from 2.7 to 2.9 V vs. *NHE* and V_red_ from −0.3 to −0.5 V vs. *NHE* [17,18]. Also, the alpha phase of iron oxide (α-Fe_2_O_3_ or hematite) is a traditional photocatalyst, with a shorter energy gap, lying from 2 to 2.3 eV, allowing the utilization of sunlight for photoexcitation and redox reactions [19,20]. More recently, new photosensitive materials have been synthesized using earth-abundant metals of low toxicity. BiVO_4_ has gained much attention due to its proper energy band gap, about 2.4 eV, suitable V_ox_ of VB and V_red_ of CB, close to 2.47 and 0.32 vs. *NHE*, respectively [21,22]. Lately, MoS_2_, a low-gap semiconductor with high surface area and tunable optoelectronic properties, has been extensively studied. Along with the morphology of the disulfide, 2D monolayer structures exhibit a direct energy gap of at least 1.9 eV, while denser and bulk structures have an indirect gap of 1.2 eV [23,24], anticipating better adsorption of target contaminants and advanced photocatalytic performance [25,26].

The aforementioned photocatalysts meet specific requirements: low cost, earth abundance, facile synthetic routes, low toxicity, high chemical stability, and high efficiency, and have been examined thoroughly in a variety of lab-scale applications [27,28]. However, poor surface area, low charge carrier density, and mobility lead to insufficient separation and ROS production, resulting in limited integration into affordable real-life applications. To overcome these shortcomings, extensive research efforts have been devoted to modifying the physicochemical properties of these materials, including the creation of heterojunctions with lower band gap semiconductors [29,30]. A plethora of innovative materials, such as g-C_3_N_4_, CQDs, COFs, TMPs, and MXenes, have been synthesized and coupled with photocatalysts to form heterostructures.

**Figure 2:**
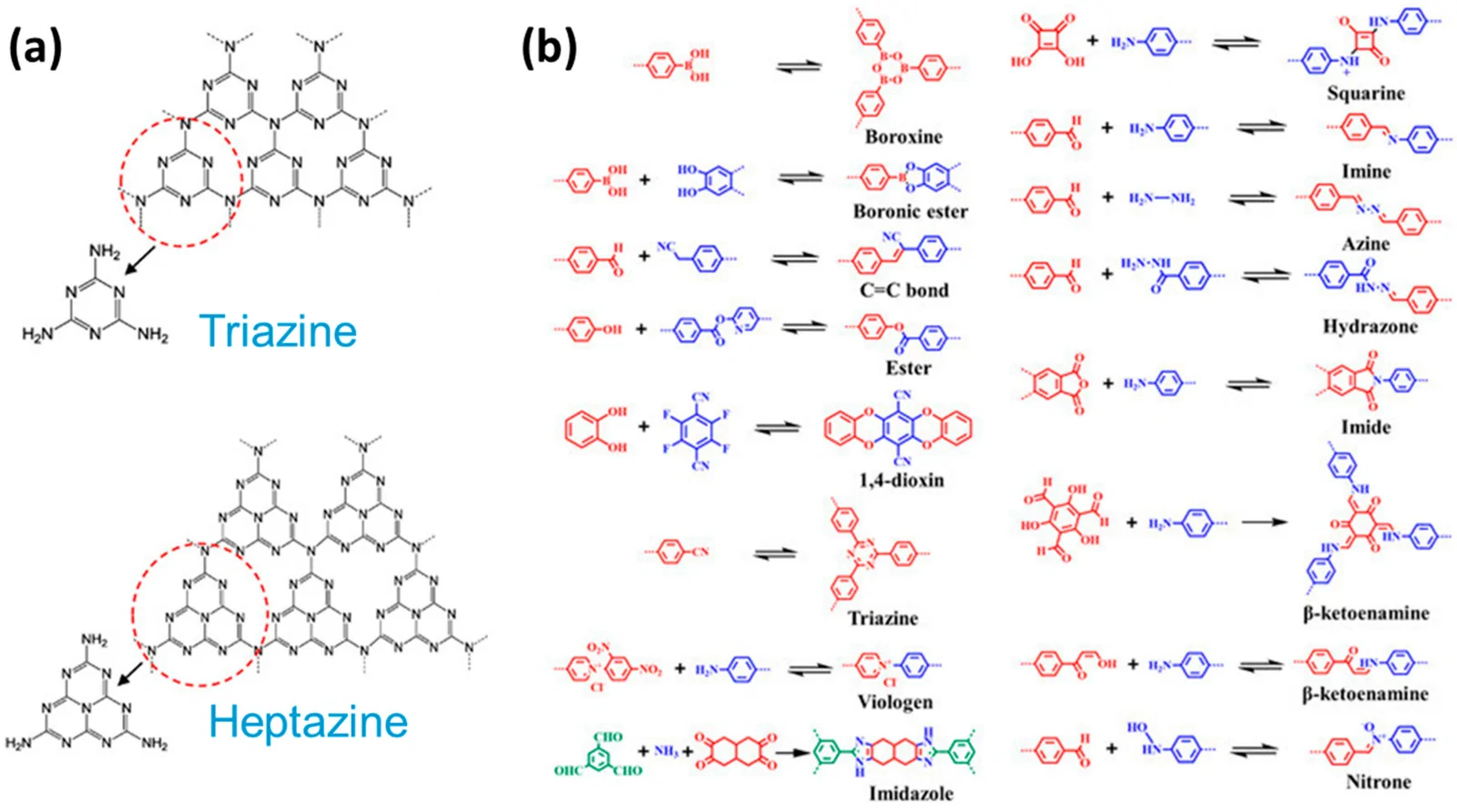
(**a**) Thermal polycondensation of heptazine and triazine for the preparation of g-C_3_N_4_. Reprinted from Ref. [31] and used under Creative Commons CC-BY license. Copyright 2025, MDPI; (**b**) Reactions of monomers for the synthesis of specific building blocks and COFs. Reprinted from Ref. [42] and used under Creative Commons Attribution CC-BY license. Copyright 2023, MDPI.

The layered g-C_3_N_4_ is a metal-free polymeric network of heptazine or triazine rings, fabricated via thermal polycondensation (Figure 2a) [31,32], with high chemical stability, high surface area, and an energy band gap close to 2.7 [33]. Even if the standalone g-C_3_N_4_ suffers from low charge carrier mobility, it has gained attention because its composites have enhanced optoelectronic and photocatalytic performance [34,35]. CQDs are alternative 0D carbon-based nanomaterials with low-cost, low-toxicity, high stability, and tunable optical response [36]. The high electrical conductivity of CQDs is advantageous for photocatalytic applications when coupled with other photocatalysts, because they can act as an electron sink, boosting the separation of charged pairs and the formation of reactive radicals [37,38]. Moreover, the introduction of different functional groups during the synthesis of CQDs can influence their absorption and electronic properties [39,40]. Other desirable candidates for the fabrication of heterostructured photocatalysts are the COFs, due to their high surface area, high chemical stability, and promising optoelectronic properties [41]. The linked building blocks of COFs are fabricated by the reaction of specific monomers (Figure 2b) [42], while the COF composites are formed through the covalent bonding of 2D or 3D crystalline polymeric networks with other materials [43]. This continuous structure of the final composite will eventually present enhanced electrochemical properties, charge carrier separation, and ROS formation [44,45].

**Figure 3:**
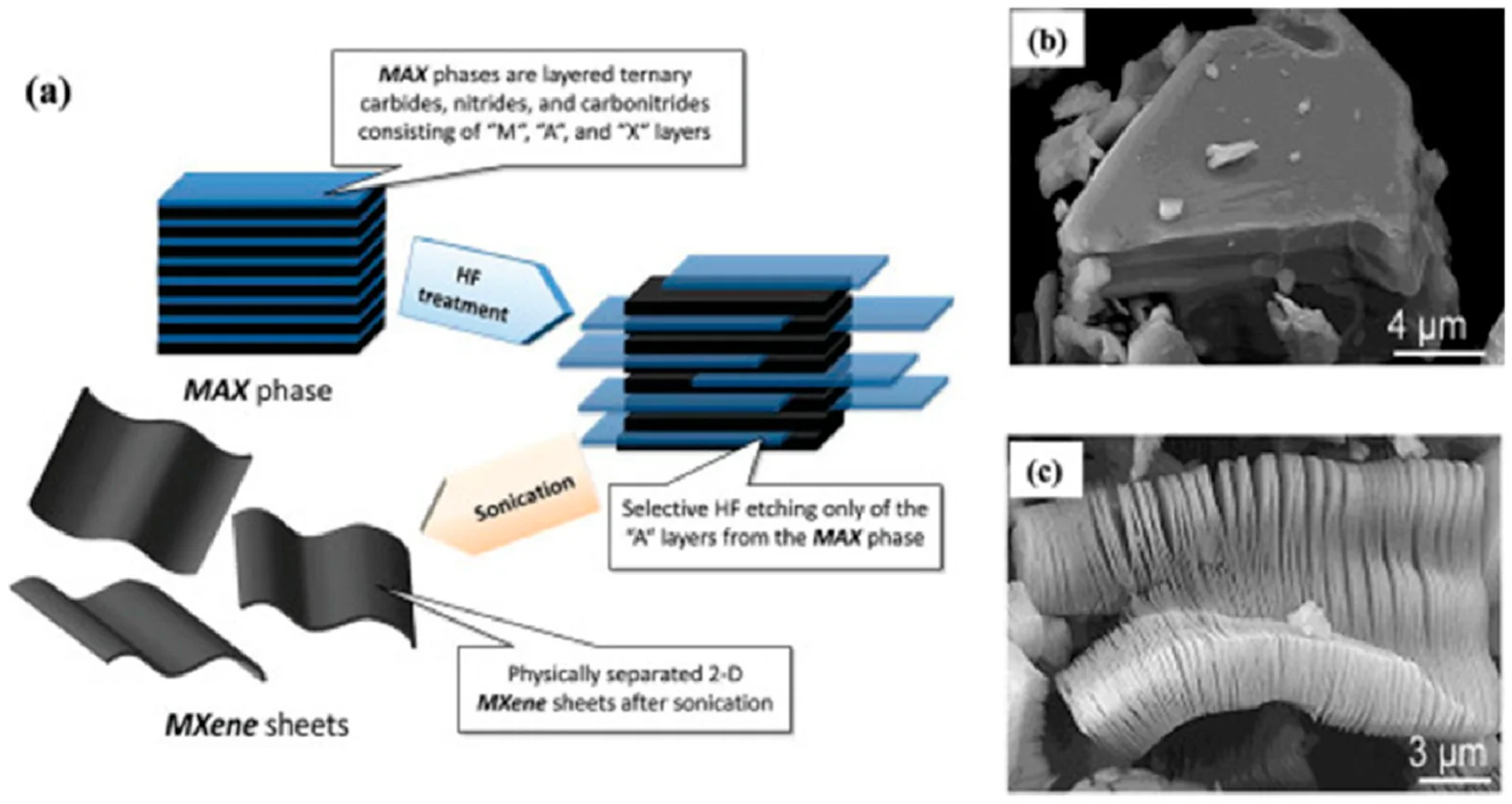
(**a**) Exfoliation of different MAX_n_ parent phases for the formation of M_n+1_X_n_T_x_; (**b**) Representative Ti_3_AlC_2_ phase before exfoliation and (**c**) after 10% HF etching. Reprinted from Ref. [58] and used under Creative Commons Attribution CC-BY 4.0 license. Copyright 2024, Elsevier.

Apart from heterostructures with carbonaceous and organic materials, the inorganic TMPs and MXenes are investigated extensively, due to their beneficial physicochemical properties [46,47]. The TMPs are inorganic crystalline compounds formed by a metal element (M) and phosphorus (P), packed into triangular prisms with M at the corners and P at the center for P-rich TMPs, or into tetrakaidecahedral arrangements for M-rich TMPs [48]. TMPs are very effective in photoinduced reactions due to the abundance of active sites on their surface, which promote H^+^ and CO_2_ adsorption [49,50], and their tunable electronic properties. Regarding the TMPs’ conductivity, the high electronegativity of phosphorus (P) and the adjustment of the M to P ratio indicate the electronic properties of TMPs [51], resembling semiconducting behavior for P-rich TMPs, with a tunable band gap for e^−^ excitation, or a metallic nature for M-rich TMPs, promoting charge carrier separation [51]. On the other hand, MXenes are 2D layered nanomaterials, with a structure similar to graphene [52]. Their general formula is M_n+1_X_n_T_x_, where M is a transition metal of the early groups of the periodic table, X represents C or N in most cases, T_x_ is a surface functional group (ex -OH, -O, -Cl), and n is the number of layers, ranging from 1 to 3 [53,54]. MXenes are fabricated by exfoliating an MAX parent phase of a specific carbide, nitride, or carbonitride under a selective HF or HF-free etching method [55,56]. In principle, a layer of A elements (belonging to the 13th or 14th group) is weakly bonded between the covalently bonded M-X unit layers of MAX, which is removed during etching, resulting in the characteristic graphene-like structure (Figure 3) [53,57,58]. MXenes are desirable for heterostructure formation due to their high surface area, the controllable interlayer spacing, and functionalization of the outer surface. Therefore, efficient e^−^/h^+^ pair separation and enhanced ROS formation can be facilitated if photocatalysts are coupled with MXenes, owing to the latter’s tunable electrical conductivity.

## 3. Heterojunctions

**Figure 4:**
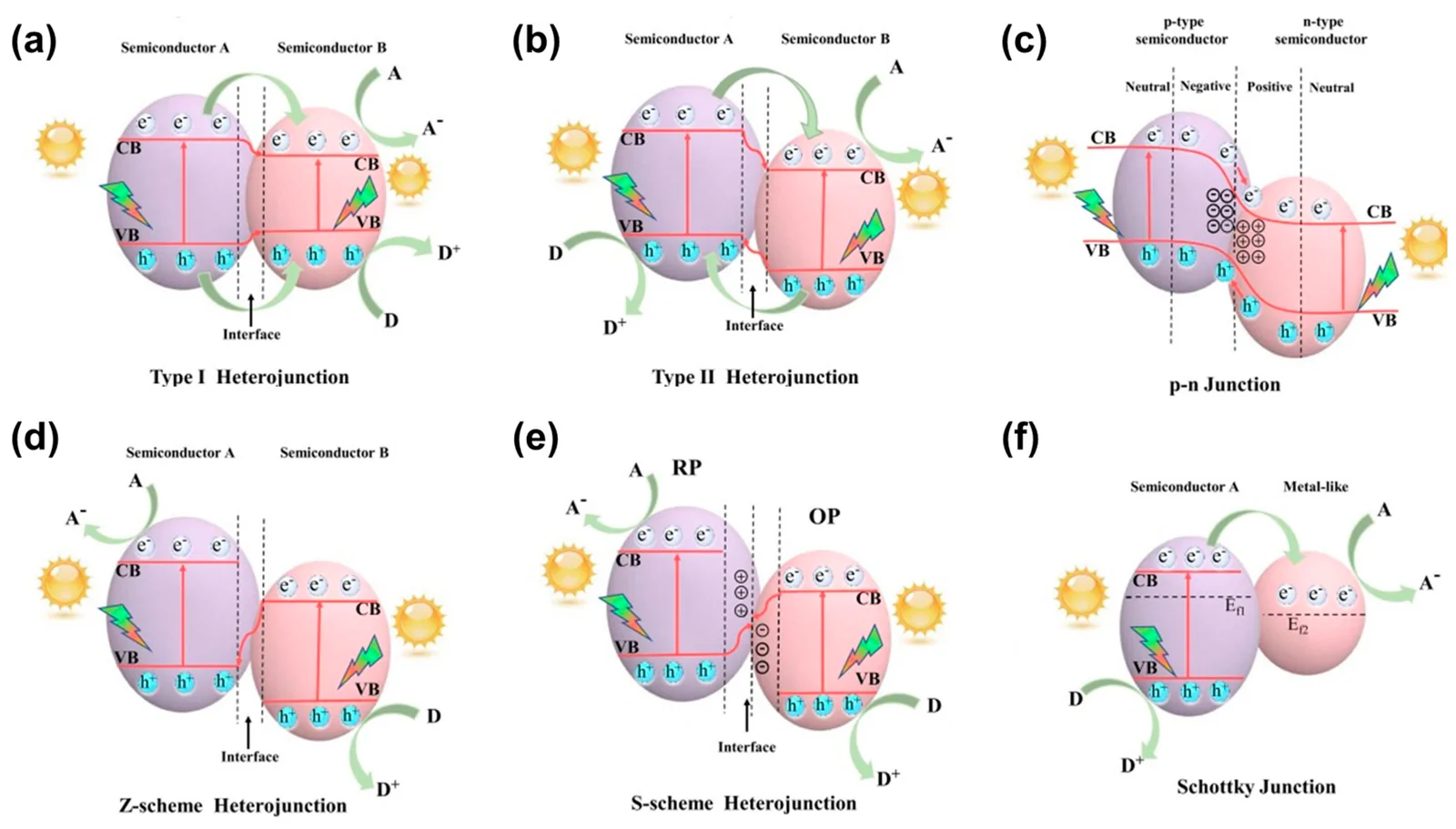
The proposed Type I (**a**); Type-II (**b**); p-n (**c**); Z-Scheme (**d**); S-Scheme (**e**); and Schottky (**f**) hetero junctions differ in the CB and VB energy levels of the two coupled semiconductors S_A_ and S_B_, which influence the photogenerated charge-pair migration pathways at the interface. Reprinted from Ref. [61] and used under Creative Commons Attribution CC-BY 4.0 license. Copyright 2024, Elsevier.

In general, heterostructure engineering involves the coupling of two semiconductors S_A_ and S_B_, with the aim of improving the charge carriers’ separation and suppressing e^−^/h^+^ recombination of the resulting composite. Depending on the corresponding VB and CB levels of each semiconductor, different types of heterojunctions can be formed, thereby altering the charge carrier transfer pathways and the ROS generation mechanism (Figure 4) [59,60,61]. In order to confirm the improved separation at the interface, complete electrochemical characterization is required, including photo-transient current [62], Mott-Schottky [63], and impedance techniques [64], whereas other methods, like fluorescence photoluminescence (PL) [65] or computational modeling and simulations [66], are also applied occasionally. Furthermore, it is possible to identify and suggest the fabricated heterosystem between S_A_ and S_B_ if CB and VB levels are determined either theoretically or experimentally using data derived from Mott-Schottky plots and absorption spectra [67,68]. Regardless of the calculation method, the energy band levels of the various composites are provided at the end of the section in a detailed table.

### 3.1. Type I

When a semiconductor S_A_ is coupled with a semiconductor (S_B_) of less negative CB and less positive VB, a Type I heterojunction is formed (Figure 4a). Irradiation of such systems with light of appropriate wavelength involves the excitation of SCs and the migration of photogenerated electrons and holes of S_A_ to the CB and VB of S_B_, respectively [59,69]. However, accumulation of e^−^/h^+^ pairs in a semiconductor with a lower redox potential is considered impractical for chemical reactions, so other types of junctions are preferred. Nevertheless, a type-I heterosystem was proposed to explain the increased evolution of H_2_ from cobalt phosphide (CoP) decorated with CTF-COF, as well as the findings of spectroscopic and electrochemical examination [70].

### 3.2. Type II

Type II heterojunction involves two semiconductors S_A_ and S_B_, whose band levels differ; the CB of S_B_ is less negative than that of S_A_, and the VB of S_B_ is more positive than that of S_A_ (Figure 4b) [59,71,72]. When the semiconductors are excited, the photogenerated electrons and holes are separated according to the potential difference of the corresponding bands. In particular, the electrons of S_A_ migrate to the less negative CB of S_B_ (CB_2_), while the holes of S_B_ follow the opposite direction, reaching the VB of S_A_ (VB_1_). As long as the redox potentials of CB_2_ and VB_1_ are appropriate, the active radicals are formed.

The construction of type II heterojunctions is a very common strategy for fabricating composites with improved photoresponse and photocatalytic performance. For instance, the Mott-Schottky analysis of TPBPD-COF with graphene-phase carbon nitride (CNNS) revealed that the CB of the components were −0.79 V vs. *NHE* for TPBPD-COF and −1.52 V vs. *NHE* for CNNS [65]. Based on diffuse reflectance spectra, the corresponding VB were estimated to be 1.56 and 1.19 V vs. *NHE*; thus, the formed heterosystem can only be classified as a type-II heterojunction. Similar analysis for 3D MoS_2_ nanoflower with a 2D TPPy-COF framework, along with results from cyclic and linear sweep voltammetry, impedance analysis, photocurrent transient, and time-resolved fluorescence, indicated that the photoexcited electrons migrate to the less negative CB of MoS_2_ and the holes to the VB of COF [73]. Combinations of COFs with layered MXene structures [74], or particulate BiVO_4_ semiconductors [71], also explained the improved charge transport due to the formation of a type II heterojunction. Other studies suggested that a type II heterojunction explains the greater removal of antibiotics, including the sulfamethoxazole contaminant, from a Cobalt Phosphide-promoted Bismuth Vanadate catalyst [75], or the increase in photocurrent by about 7.5 times when a hematite film was deposited onto TiO_2_ nanoparticles [76].

### 3.3. p-n Junction

The e^−^/h^+^ transfer pathways in p-n junctions are similar to those of type II, though the p-type conductivity of S_A_ and the n-type of S_B_ are vital for the separation mechanism. When S_A_ and S_B_ come in contact, a potential difference occurs, driving the electrons from S_A_ and the holes from S_B_ to migrate at the interface. Consequently, the interface region of S_A_ becomes negatively charged, while that of n-type S_B_ becomes positively charged. Once the Fermi levels of the semiconductors align, a Built-In Electric Field (BIEF) is established, directed from S_B_ to S_A_. Thus, the photogenerated e^−^/h^+^ pairs will be able to overcome this BIEF, with a direction proportional to their charge, and become separated (Figure 4c) [59,77,78]. Such an efficient heterojunction was fabricated by Cheng et al., using p-type MoS_2_ microspheres and n-type BiVO_4_ nanoparticles, achieving approximately 14 times higher first-order kinetic constants for the photodegradation of tetracycline hydrochloride [79].

### 3.4. Z-Scheme

A comparable system to Type II is the direct Z-Scheme system, where the involved energy bands of S_A_ and S_B_ resemble those of Type II (Figure 4d). However, in this case the redox potentials favor the recombination of photoinduced e^−^ from S_B_ with h^+^ of S_A_ [59,80]. Compared to the type-II system, the remaining e^−^ accumulate in the CB of S_A_, which possesses the highest available reduction capacity, while the h^+^ accumulate in the VB of S_B_, which exhibits stronger oxidation capacity. Consequently, the Z-scheme system overcomes the redox power limitations of type II, in which the charge carriers migrate in the opposite direction, resulting in a lower overall redox capacity [72].

**Figure 5:**
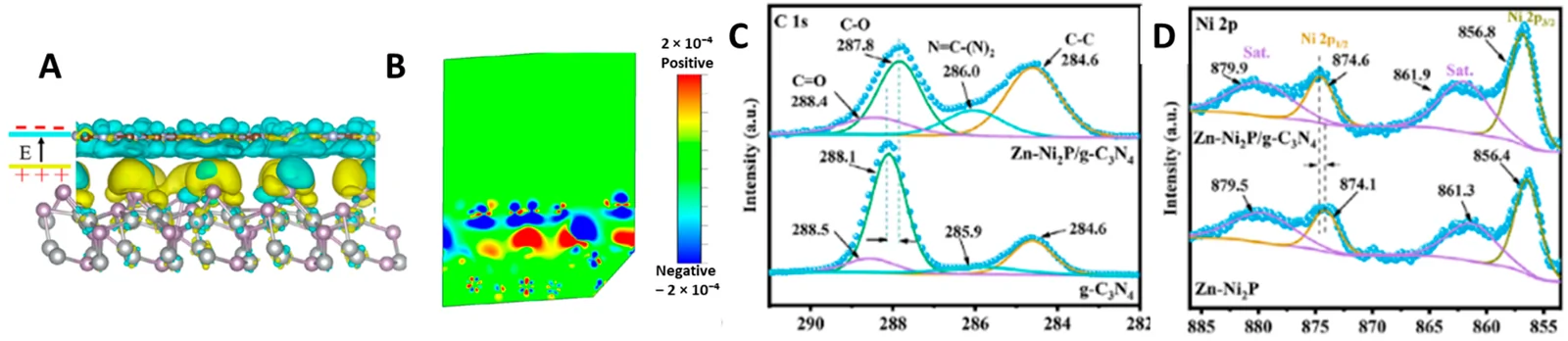
(**A**) Differential charge density of Zn-Ni_2_P/g-C_3_N_4_, where negative charges (in yellow) gather in Zn-Ni_2_P, and positive charges gather in g-C_3_N_4_ (cyan); (**B**) the corresponding longitudinal section image of the composite; (**C**) Indicative C 1s and (**D**) Ni 2p core-level XPS spectra of g-C_3_N_4_ and Zn-Ni_2_P/g-C_3_N_4_. Reprinted from Ref. [83] and used under Creative Commons Attribution CC-BY 4.0 license. Copyright 2025, Wiley-VCH GmbH.

In this context, the BiVO_4_ photoelectrons were shifted towards the VB of MoS_2_, demonstrating better charge transfer for the MoS_2_/BiVO_4_ composite due to lower interfacial resistance and less energy loss, as further supported by Electron Paramagnetic Resonance (EPR) analysis and Density Functional Theory (DFT) simulations [66]. In particular, the theoretically calculated values of E_g_, Fermi levels (E_F_), and work functions (Φ) for MoS_2_ and BiVO_4_ indicated electron flow at the contact interface, which is in agreement with the calculated CB and VB, based on the empirical formulas of Mulliken’s electronegativity Theory [66]. Likewise, it was suggested that micro- and macroporous triazine-COFs grown on amino-modified BiVO_4_ nanosheets follow a Z-scheme charge separation pathway, after analyzing Mott-Schottky plots and monochromatic photocurrent action spectra (MPAS) [81]. The participation of carbon nitride in Z-Scheme systems with Ni_2_P was experimentally evidenced after determining its CB and VB positions with Mott-Schottky plots and XPS valence band spectra, respectively [82]. In another study on Zn-Ni_2_P/g-C_3_N_4_ composites, the results of the theoretically predicted differential charge density were correlated with the observed lower C 1s and N 1s binding energies and the upshifted binding energies of Ni 2p, Zn 2p, and P 2p, the improved charge carrier separation and PL properties, to indicate the formed heterosystem (Figure 5) [83]. Lastly, theoretical calculations of energy band positions predicted that the combinations of carbon nitride with Fe_2_O_3_, BiVO_4_, and MoS_2_ could also function as a Z-scheme system [64,84,85].

However, it should be mentioned that the direct Z-Scheme cannot adequately explain the charge carrier migration, as it deviates from the principles of conventional and all-solid-state Z-Schemes; thus, the S-Scheme heterojunction system is suggested [86].

### 3.5. S-Scheme

In the case of the more complex S-Scheme systems, the coupled semiconductors should be selected regarding their redox potential [77,86]. The first semiconductor should have a strongly oxidizing VB, with a potential more positive than that required for the O_2_/H_2_O oxidation reaction, and an almost positive CB level that is unable to activate reduction reactions. On the other hand, the CB of the second semiconductor should correspond to very negative potentials, enabling reduction reactions, while its holes should have a low V_ox_. The heterojunction between the so-called oxidation (S_O_) and reduction semiconductor (S_R_) leads to the alignment of their Fermi levels, the bending of their band levels, and the formation of an internal electric field at the interface (Figure 4e) [59,77,87]. The e^−^ in the CB of S_O_ and the h^+^ in the VB of S_R_ migrate toward the charged interface, under the influence of the internal electric field, where they recombine. Hence, the strongly oxidizing h^+^ in S_O_ and the reducing e^−^ in S_R_ are preserved, initiating redox reactions.

To date, many studies have proposed that many COFs act as S_R_ when contacted with ZnO [88,89]. For example, Mu et al. conducted Ultraviolet Photoelectron Spectroscopy (UPS) to calculate the energy bands, in situ XPS to detect both BIEF and band bending, and finally in situ EPR spin trapping experiments to rule out a type II system between the N-ZnO and TAPT-DMTP COF [89]. They concluded that the S-Scheme indeed applies to the examined composite, and verified that the optimal production of hydrocarbons comes from the lower charge transfer resistance and the faster electron transfer rate of the N-ZnO/TAPT-DMTP COF composite. Other indicative and comprehensive studies on S-Scheme heterosystems provided either experimental evidence on the interfaces of a carbon nitride sponge containing BiVO_4_ [90], or theoretical ab initio calculations for a MoS_2_ monolayer coupled with boron phosphide [91].

### 3.6. Schottky Junction

**Figure 6:**
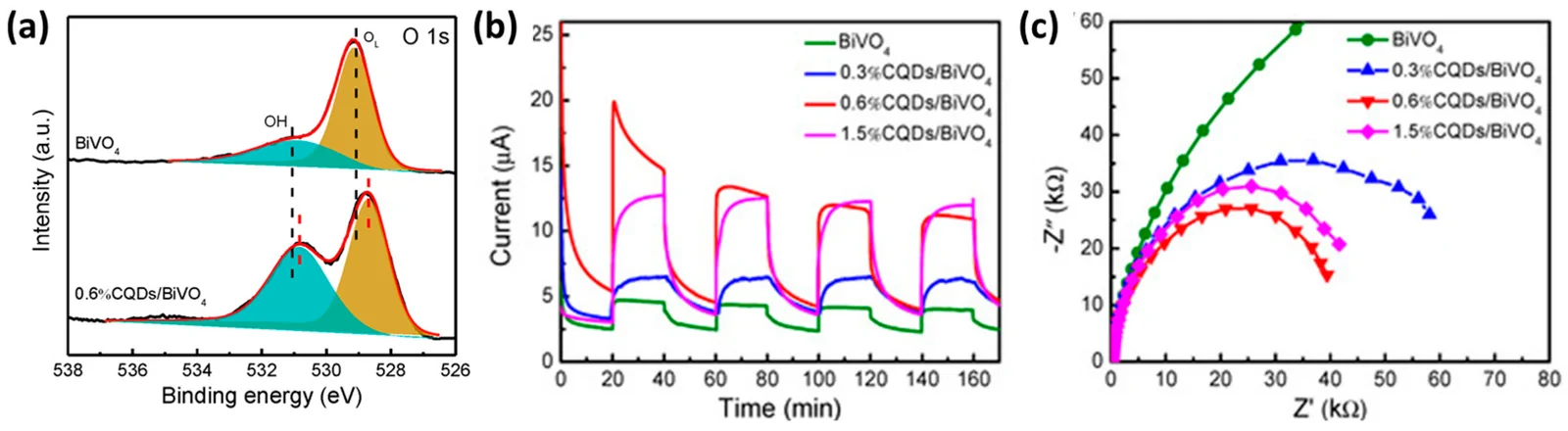
(**a**) O 1s core level XPS spectra; (**b**) photocurrent transient; and (**c**) Nyquist impedance plot of CQDs/BiVO_4_. Reprinted from Ref. [94] used under Creative Commons Attribution CC-BY license. Copyright 2023, MDPI.

Sometimes, the aforementioned models cannot describe the electronic properties and the charge transfer mechanism at the heterojunction of a composite, although electrochemical and spectroscopic examination validates that one of the components acts as an electron sink. This limitation is very typical for highly conductive nanomaterials, especially for carbon dots, metal phosphides, and MXenes [92,93,94,95]. Such an example is the enhanced transient photocurrent response and the reduced interfacial charge transfer resistance of BiVO_4_ and carbon quantum dots, where XPS reveals the chemical interaction, but the aforementioned heterosystems cannot be applied (Figure 6) [94].

In many cases, the model of a Schottky junction can be applied, which generally describes the coupling of a semiconductor with metal elements of lower Fermi level, e.g., silver nanoparticles [29], and the photoexcited electrons are shifted towards the metal until the Fermi levels of the metal and the SC reach equilibrium (Figure 4f) [59,77]. In this context, Liu et al. suggested that the lower binding energies in the O 1s core level XPS spectra of the ZnO@Ti_3_C_2_ composite, in combination with the prolonged increase in photocurrent, the reduced surface resistance, PL intensity, and fluorescence lifetime, demonstrated the formation of a Schottky junction [95]. In a different approach, the evaluation of the DFT calculations and the experimental results of XPS, PL, photocurrent, and electrochemical impedance analysis displayed the formation of the junction on a porous graphitic carbon nitride (PGCN) network decorated with Ti_3_C_2_ quantum dots [96].

In conclusion, Table 2 below provides a detailed summary of all the aforementioned heterostructures, the resulting heterosystems and the calculated energy band levels, as well as the synthesis methods for each component and composite.

## 4. Photocatalytic Oxidation and Reduction Processes

### 4.1. Pollutant Removal

Nowadays, environmental remediation with photocatalytic materials is extensively investigated, especially for the degradation of persistent contaminants in water and air, including harmful pathogens. As described earlier, photocatalytic oxidation processes are based on the formation of reactive and non-selective ROS, due to their high V_ox_ and high kinetic reaction constants, which range from 10^6^ to 10^9^ M^−1^ s^−1^ [97,98]. The heterostructured composites are expected to improve the overall performance of a photocatalytic system, since the separation of e^−^/h^+^ pairs in the junction suppresses any undesired recombination effects and enhances ROS formation. Even if the type of heterojunction remains unclear in some studies, the photocatalytic performance of the selected heterostructures in eliminating organic substances, such as pharmaceuticals, dyes, and raw ingredients, different types of microorganisms, or even inorganic compounds and heavy metal ions, is indisputable. Detailed tables at the end of each subsection summarize the reported composites in the literature, their properties (if available), and the experimental conditions applied, allowing for a direct comparison of their photocatalytic performance.

#### 4.1.1. Organic Substances

**Figure 7:**
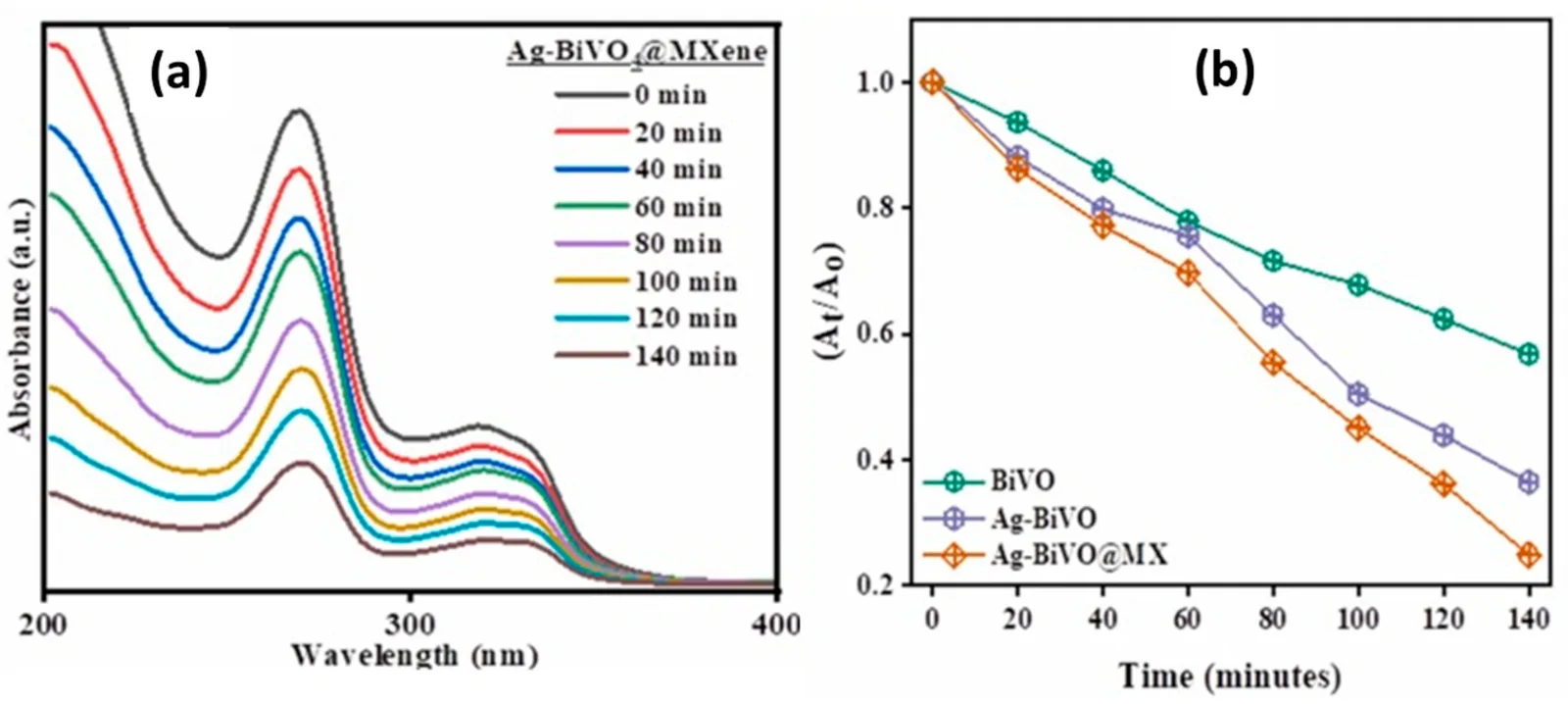
(**a**) Reduction of the characteristic absorption peak of ciprofloxacin with time, in the presence of Ag-BiVO_4_@MXene; (**b**) Degradation kinetics of ciprofloxacin and the corresponding reference materials BiVO and Ag-BiVO. Reprinted from Ref. [103] used under Creative Commons Attribution CC-BY 4.0 license. Copyright 2025, Elsevier.

The removal of antibiotics and other pharmaceuticals is of high importance, as the accumulation of such substances in groundwater and surface water poses potential risks for human health [99,100,101]. In this regard, Ge et al. managed to degrade 99% of acetaminophen, a very common analgesic, after one hour using the Heptazine-COF@TiO_2_ composite [102]. They proved that the composite had higher rate constants than the stand-alone catalysts, with reproducible removal efficiencies after six recycling experiments, due to the e^−^/h^+^ separation under a type-II system. The photodecomposition of another non-biodegradable antibiotic, ciprofloxacin, was extensively examined using different photocatalysts [103,104,105,106]. Ahmed et al. monitored the compound’s concentration with a UV/vis spectrophotometer and distinguished the degradation kinetics of Ag-BiVO_4_@MXene (Figure 7) [103]. In a similar study, the kinetics and the possible degradation pathways of ciprofloxacin were assessed by LC-MS methods, while the optimal ratio of ZnO to MoS_2_ composite and the effect of catalyst dosage were also determined [104]. Alternative materials, including carbon quantum dots modified g-C_3_N_4_ nanotubes onto a carbon cloth substrate and BiVO_4_@g-C_3_N_5_ core-shell heterostructure, have also been tested for the removal of ciprofloxacin [105,106]. Lastly, the antibiotics cefixime and clindamycin were used as model contaminants to evaluate the pairing of carbon nitride with the 2D Ti_3_C_2_ MXene and its photocatalytic activity [107,108], or the anti-inflammatory ibuprofen for a TiO_2_ inverse opal structure decorated with Mo-doped BiVO_4_ [63].

Apart from pharmaceuticals, organic dyes from aqueous solutions are widely utilized as target compounds in photocatalytic processes [109,110]. For instance, the degradation efficiency of methylene blue, a typical textile dye, was accelerated when a ZnO/V_2_C-MXene hybrid was used instead of bare ZnO nanosheets [111]. This improvement was attributed to the enlarged surface area and the interfacial migration of photoelectrons to V_2_C. Other MXene materials, like Ti_3_C_2_(OH)_2_, act as photocatalysts; thus, better removal of Malachite Green was expected when Ti_3_C_2_(OH)_2_ was integrated with Carbon Dots [112].

The removal of common industrial ingredients and solvents is also investigated, because they are hazardous if released to the environment. For example, the photocatalytic performance of PCDots-C_3_N_4_ was demonstrated using Bisphenol A, a chemical for the fabrication of polymers [62]. The preservative benzyl paraben, which can cause toxicity problems in high amounts, was removed by a BiVO_4_ decorated with Carbon Dots [94]. The degradation and mineralization of gaseous benzene presented a 2-fold enhancement when the pristine BiVO_4_ was coupled with α-Fe_2_O_3_ [113]. Also, the mineralization process of gaseous o-xylene by Ti_3_C_2_-TiO_2_ composite was studied in depth, using in situ Diffuse Reflectance Infrared Fourier Transform Spectroscopy (DRIFTS) measurements [114].

Finally, Table 3 presents in detail the experimental conditions and the photocatalytic performance of the aforementioned composite materials in the removal of pharmaceutical compounds, dyes and industrial substances.

#### 4.1.2. Sterilization and Antimicrobial Properties

Apart from organic molecules, photocatalysis can also deal with waterborne and airborne microorganisms, achieving disinfection and sterilization of water, air, and surfaces [115,116]. The exact photodegradation and elimination mechanism of living pathogens is highly dependent on the structure, the size, and the biochemical properties of the microbial load. In principle, ROS will progressively attack the outer membrane of the pathogens (if it exists) and the peptidoglycan layer, reaching the inner membrane [117,118]. If the damage to the cell wall is extensive and irreversible, then the cell permeability will cause partial release of the interior cytoplasm and leakage of intracellular substances, such as proteins and nucleic acids, and finally cell death will occur [118,119].

The antimicrobial properties of the photocatalytic materials presented are evaluated on a lab scale according to the standard plate count (Table 4), where contaminated feed is cultured in nutrient media before and after treatment [119,120,121]. This method is highly sensitive, even when the growth of gram-positive *Staphylococcus aureus* and gram-negative *Escherichia coli* was inhibited by N-doped Carbon Dots modified MoS_2_ nanosheets, captured onto a PEI-modified Ti foil [120]. Other studies followed similar procedures to validate the disinfection of *Pseudomonas aeruginosa* and *S. aureus* by a flower-like MoS_2_ covered with a BiVO_4_ layer [66], the elimination of *S. aureus* by MoS_2_ coupled with Cobalt Phosphide (CoP) nanorods [119], or the deactivation of *E. coli* by COF@CDs [121]. 

#### 4.1.3. Inorganic Substances

Photocatalysts can also deal with inorganic contaminants, mainly toxic heavy metal ions in water, or inorganic gases [122,123]. Most studies focus on the transformation of hexavalent chromium ions, Cr(VI), to the less toxic trivalent Cr(III), in order to evaluate the composite materials in advanced reduction processes [124,125]. For example, bifunctional MoS_2_/α-Fe_2_O_3_ completely removed Cr(VI), owing to lower charge transfer resistance and suppressed recombination losses [126]. A more comprehensive investigation used metal-free TPBPD-COF-carbon nitride nanosheet hybrids for the photocatalytic removal of Cr(VI) in batch and dynamic column experiments [65]. The photocurrent transient and impedance spectra, in combination with absorption and steady-state PL analysis, showed that the formed type-II heterostructure improved the charge carriers’ separation and the performance of the hybrid catalyst. Last, Table 5 presents in detail the experimental conditions and results for the aforementioned composite materials regarding the reduction of Cr(VI).

### 4.2. Production of Solar Fuels

The photoelectrocatalytic water splitting and hydrogen evolution using TiO_2_ were first investigated by Honda and Fujishima in 1972 [13]. The same group also discovered that TiO_2_ produces valuable hydrocarbons via the photoelectrocatalytic reduction of CO_2_ [127]. Since then, the field of light-driven fuel synthesis has emerged as an established research area; a plethora of new materials have been tested, and countless experimental setups have been designed.

#### 4.2.1. H_2_O Splitting and H_2_ Evolution

The photocatalytic production of hydrogen requires the presence of an aqueous medium or water vapor, while the energy bands of the SC should correspond to appropriate redox potentials [128]. Thus, when the SC is excited by light of appropriate energy (Equation (5)), the photoholes (h^+^) will break down water only if the VB corresponds to a minimum V_ox_ of 1.23 V vs. *RHE* according to Equation (6). Provided that V_red_ of CB matches the potential of the H^+^/H_2_ reaction, the photoexcited e^−^ will then promote hydrogen evolution, as described in Equation (7) [129,130].

SC + hv → e^−^ + h^+^ (5)

2 H_2_O + 4 h^+^ → 4 H^+^ + O_2_   E^0^ = 1.23 V vs. *RHE* (6)

4 H^+^+ 4 e^−^ → 2 H_2_   E^0^ = 0 V vs. *RHE* (7)

**Figure 8:**
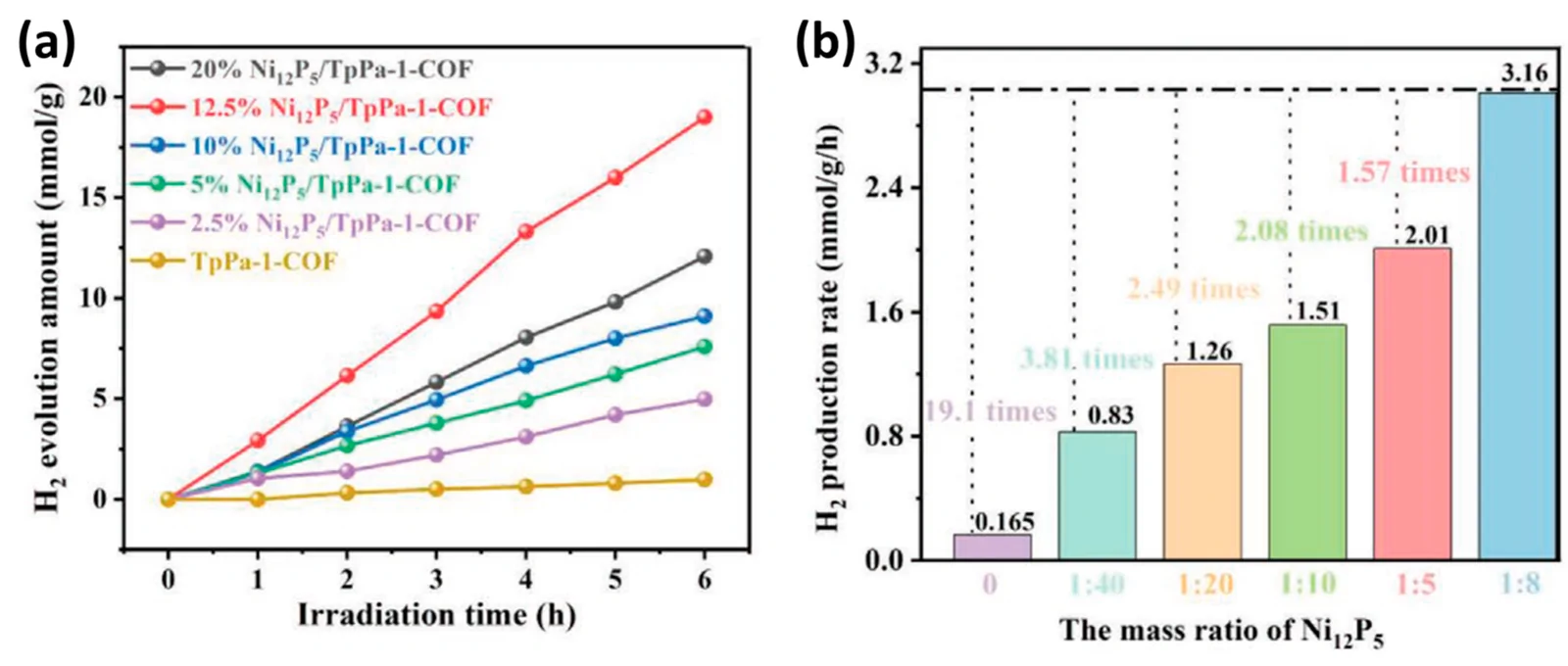
(**a**) Photocatalytic H_2_ evolution amount for the different Ni_12_P_5_/TpPa-1-COF composites with time; (**b**) Comparison of H_2_ generation rates for the different Ni_12_P_5_/TpPa-1-COF composites. Reprinted from Ref. [131] used under Creative Commons Attribution CC-BY 4.0 license. Copyright 2022, Wiley-VCH GmbH.

Additionally, the limited lifetime of the photogenerated charged pairs should also be taken into consideration because any recombination effects lower the H_2_ evolution rates. A generally accepted strategy is the deposition of highly conductive materials, such as metal phosphides and MXenes, onto photocatalysts, with the aim of enhancing the charge transport at the interface. In this context, the introduction of 12.5% Ni_12_P_5_ onto TpPa-1-COF (ratio of 1:8) exhibited more than 19 times higher hydrogen production than the parent components (Figure 8) [131]. More interestingly, Zhou et al. improved remarkably the performance of pristine TAPB-Tp-COF by almost 27 times by fabricating a stable composite, with high porosity and conductivity, through coupling with the optimal ratio of NH_2_-Ti_3_C_2_T_x_ [74]. Other effective combinations include a series of TiO_2_/NiP, TiO_2_/Ti_3_C_2_, and TiO_2_/NiP/Ti_3_C_2_ titania composites [93], or a MoS_2_/Ti_3_C_2_ photocatalyst [132]. In that study, the 30 wt % Ti_3_C_2_ loading onto MoS_2_ increased the hydrogen production rate from 2626 to 6144 μmol g^−1^ h^−1^ after 5 h of visible irradiation, owing to reduced recombination rates and the increased surface area of the final composite (Table 6) [132]. Lastly, Table 6 illustrates the experimental conditions and H_2_ production rates for the composites reported in this study.

#### 4.2.2. CO_2_ Reduction

The photocatalytic reduction of CO_2_ to hydrocarbons is a sustainable green chemistry approach to transform greenhouse emissions into valuable solar fuels. As a first step, CO_2_ gas must be transferred onto the surface of the catalyst, and then it will be transformed into simple carbon compounds, provided that the photoexcited h^+^/e^−^ have sufficient redox potential. One possible reduction pathway suggests that CO_2_ is progressively converted to formic acid (HCOOH), formaldehyde (HCHO), liquid methanol (CH_3_OH), until it forms methane gas (CH_4_) [133]. For example, the heterostructured catalyst MoS_2_@TPPy-COF achieved the production of 966 μmol g^−1^ HCOOH after 3 h, owing to the visible light activation of the composite and the improved separation of charged pairs at the interface (Table 7) [73]. An alternative mechanism starts with the reduction of CO_2_ to carbon monoxide (CO), and then CH_3_OH and CH_4_ are formed [134]. In this regard, Lin et al. focused on CO evolution using a carbon-dot-modified 0D-ZnO catalyst, whose CO yields were close to 119μmol g^−1^ h^−1^, surpassing the 32.2 μmol g^−1^ h^−1^ of pristine 0D-ZnO and 5.3 μmol g^−1^ h^−1^ of Degussa TiO_2_ (Table 7) [135].

COF-based composites have gained considerable attention for CO_2_ photoconversion, because they present better adsorption capacities and higher apparent quantum yields. For instance, N-ZnO/TAPT-DMTP COF hybrid surpassed the activity reported for its individual components, producing 11.07 μmol g^−1^ of CO and 4.86 μmol g^−1^ of CH_4_ after 5 h (Table 7) [89]. In another case, triazine-COF (IL@MTPA) was grown on amino-modified BiVO_4_ nanosheets and then confined with the [EMIM][NTf_2_] ionic liquid, in order to fabricate a mesopore-nanoreactor [81]. The optimal composite resulted in CO_2_ conversion rates of 903.6 μmol g^−1^ h^−1^, with nearly 100% selectivity to CO and CH_4_ products, highlighting a 6-fold enhancement compared to the non-confined composite and a 38-fold enhancement compared to the BiVO_4_ catalyst. Remarkably, the study of a synthesized MoS_2_@COFs hybrid composite revealed high selectivity in producing up to 56.2 μmol g^−1^ h^−1^ of the heavier and highly desirable ethane (C_2_H_6_), instead of releasing only CO, H_2_, and CH_4_ as the main products (Table 7) [134]. Additionally, all intermediates and final products, CO, CH_4_, and C_2_H_6_, were detected using DRIFTS, indicating the whole reaction pathway.

## 5. Summary and Future Research

The stand-alone TiO_2_, ZnO, Fe_2_O_3_, BiVO_4_, and MoS_2_ materials are recognized as the most efficient photocatalysts for the degradation of organic and inorganic substances or the production of solar fuels. Even so, intense efforts are constantly devoted to improving the charge carriers’ separation and the overall photocatalytic activity. The coupling of these materials with carbon-based materials, like carbon nitride, carbon dots, and COFs, or inorganic materials, like TMPs and MXenes, has gained great attention because the fabricated heterostructures present unique electronic properties. Depending on the formed heterojunction system—type I, type II, Z-scheme, S-scheme, Schottky junction—the excitation and migration mechanism of the photogenerated e^−^/h^+^ pairs alters among the different composites, leading to better charge carrier mobility and performance in photoinduced applications. This review reports most binary combinations, highlighting their effectiveness in the photodegradation of emerging contaminants, including pharmaceuticals, industrial ingredients, and pathogenic microorganisms, as well as the generation of H_2_ and hydrocarbons. However, numerous challenges remain to be addressed, and many opportunities for utilization of these materials remain unexplored.

The main challenge for these photocatalytic composites is long-term stability, which concerns both the stability of the individual components and the stability of the interface. Although TiO_2_ and carbon-based materials are considered highly stable, the same does not apply to the other materials. MXenes and ZnO may undergo photocorrosion in the presence of water under UV illumination, leading to metal leaching and deterioration of their photocatalytic performance. Materials like MoS_2_, BiVO_4_, and TMPs are photostable under neutral conditions, though they are also susceptible to chemical dissolution in strongly acidic or alkaline environments. Apart from improving optoelectronic properties, the fabrication of heterostructures also serves as an excellent approach for enhancing the stability of specific materials. The migration of charge carriers toward the stable component can suppress undesired chemical reactions and structural breakdowns within the other component. Still, interfacial stability and long-term performance should be investigated using spectroscopic techniques and long-term cycling experiments, respectively, in order to confirm that photocorrosion or surface contamination is prevented and that the efficacies are maintained over time.

Another significant challenge for heterostructured photocatalysts concerns the scaling-up of applications, as well as their use under real-world conditions. Most studies on pollutant degradation are conducted using particular model compounds, avoiding real effluent or real water matrices. Moreover, most studies address the removal of pollutants solely in terms of concentration reduction, without reporting the formation of any undesired and highly toxic byproducts or the mineralization rates of the parent compounds. Similarly, the production of solar fuels is examined on the lab scale, without considering the technical challenges involved in scaling up application, nor the balance between product output and economic costs. Researchers should focus on these gaps, as existing technologies are increasingly adopting photocatalytic processes, while the reported heterostructured photocatalysts exhibit high efficiencies and represent potential materials of choice. Furthermore, particular protocols must be established, enabling direct and independent comparison of the different materials, provided that specific parameters, such as pollutant concentration, catalyst dosage, environmental conditions, and light source irradiation, are harmonized.

## Figures and Tables

**Table 1:** Binary heterostructures of metal oxides, phosphides, MXenes, and carbon-based materials ^1,2^.

	ZnO	α-Fe_2_O_3_	BiVO_4_	MoS_2_	g-C_3_N_4_	COFs	CQDs	TMPs	MXenes
**TiO_2_**	✔	✔	✔	✔	✔	✔	✔	✔	✔
**ZnO**		✔	✔	✔	✔	✔	✔	✔	✔
**α-Fe_2_O_3_**			✔	✔	✔	✔	✔	✔	✔
**BiVO_4_**				✔	✔	✔	✔	✔	✔
**MoS_2_**					✔	✔	✔	✔	✔
**g-C_3_N_4_**						✔	✔	✔	✔
**COFs**							✔	✔	✔
**CQDs**								✔	✔
**TMPs**									✔

^1^ Data retrieved from the Web of Science platform by limiting the timeframe to the last 5 years. ^2^ Binary composites discussed in the present review are marked in green.

**Table 2:** Heterosystem and energy band levels of the composite materials.

Heterostructure	Junction	Band Gap/Band Positions	Synthesis Method	Ref.
CoP decorated CTF-COF	I	CB_CTF_ = −0.74 V vs. NHE CB_CoP_ = −0.4 V vs. NHE VB_CTF_ = 1.69 V vs. NHE VB_CoP_ = 1.3 V vs. NHE	As synthesized CoP COF decoration: In situ self-assembly method, followed by a calcination treatment	[70]
COFs with BiVO_4_	II	CB_COF_ = −0.43 V vs. NHE CB_BiVO4_ = −0.57 V vs. NHE VB_COF_ = 1.94 V vs. NHE VB_BiVO4_ = 1.82 V vs. NHE	BiVO_4_: reflux-assisted synthesis, followed by thermal annealing COFs: Solvothermal Schiff base condensation reaction	[71]
TPBPD-COF-g-C_3_N_4_ (PGCN)	II	CB_TPBPD_ = −0.79 V vs. NHE CB_CNNS_ = −1.52 V vs. NHE VB_TPBPD_ = 1.56 V vs. NHE VB_CNNS_ = 1.19 V vs. NHE	CNNS: Thermal polymerization of dicyandiamide TPBPD-COFs: Schiff base reaction	[65]
MoS_2_ @ TPPy-COF	II	Direct E_g_ MoS_2_@TPPy-COF = 1.98 eV Indirect E_g_ MoS_2_@TPPy-COF = 1.8 eV	Pre-synthesized TPPy-COF MoS2@TPPy-COF: hydrothermal process	[73]
TAPB-Tp COFs with MXenes	II	E_g TAPB-Tp COF_ = 2.32 eV E_g MXene0.5/COF_ = 2.39 eV CB_TAPB-Tp COF_ = −0.478 eV CB_MXene0.5/COF_ = −0.624 eV	MXene: Ti_3_AlC_2_ etching dissolved LiF in HCl TAPB-Tp COFs: Schiff base reaction	[74]
α-Fe_2_O_3_ film onto TiO_2_ NPs substrate	II	E_g_ α-Fe_2_O_3_ = 2.04 eV E_g_ TiO_2_ = 3.35 eV	TiO_2_ on FTO substrate α-Fe_2_O_3_: SILAR method	[76]
MoS_2/_BiVO_4_	p-n	CB_MoS2_ = −0.175 eV CB_BiVO4_ = −0.505 eV VB_MoS2_ = 1.465 eV VB_BiVO4_ = 2.185 eV	MoS_2_: hydrothermal process BiVO_4_: microwave-assisted synthesis	[79]
g-C_3_N_4_ with α-Fe_2_O_3_	Z-scheme	CB_α-Fe2O3_ = 0.42 V vs. NHE CB_g-C3N4_ = −1.15 V vs. NHE VB_α-Fe2O3_ = 2.36 V vs. NHE VB_g-C3N4_ = 1.48 V vs. NHE	α-Fe_2_O_3_: as prepared α-Fe_2_O_3_/g-C_3_N_4_: in situ polymerization in the presence of the oxide	[64]
MoS_2_/BiVO_4_	Z-scheme	CB_MoS2_ = 0.105 eV CB_BiVO4_ = 0.29 eV VB_MoS2_ = 1.555 eV VB_BiVO4_ = 2.79 eV	MoS_2_: hydrothermal process Composite: in situ growth of BiVO_4_ under hydrothermal process	[66]
IL@MTPA/BiVO_4_	Z-scheme	CB_BVO_ = 0.33 V vs. NHE CB_COF-MPA_ = −0.55 V vs. NHE CB_COF-MPTA_ = −0.60 V vs. NHE VB_BVO_ = 2.61 V vs. NHE VB_COF-MPA_ = 1.28 V vs. NHE VB_COF-MPTA_ = 1.27 V vs. NHE	NH_2_-BVO: solvothermal route COF-MPA and COF-MTPA: condensation reaction MPA/BVO and MTPA/BVO: controlled in situ growth method	[81]
Zn-Ni_2_P/g-C_3_N_4_	Z-scheme	E_g Zn-Ni2P_ = 2.02 eV E_g gC3N4_ = 2.74 eV VB_Zn-Ni2P_ = −0.34 eV VB_g-C3N4_ = 2.41 eV	Zn-NiP: hydrothermal synthesis Zn-NiP/gC_3_N_4_: hydrothermal synthesis in the presence of g-C_3_N_4_	[83]
g-C_3_N_4_ (PCN) with MoS_2_	Z-scheme	CB_PCN_ = −1.05 eV CB_MoS2_ = 0.41 eV VB_PCN_ = 1.69 eV VB_MoS2_ = 1. 84 eV	PCN: heating polycondensation method MoS_2_: hydrothermal method	[85]
COFs with ZnO	S-scheme	CB_TpPa-Cl_ = −0.72 V vs. NHE CB_ZnO_ = −0.33 V vs. NHE VB_TpPa-Cl_ = 1.23 V vs. NHE VB_ZnO_ = 2.75 V vs. NHE	TpPa-Cl COF: template-free solution-based aging method ZnO: precipitation method, followed by thermal calcination method	[88]
N-ZnO/TAPT-DMTP COF	S-scheme	CB_N-ZnO_ = −0.28 V vs. NHE CB_TAPT-DMTP COF_ = −0.68 V vs. NHE VB_N-ZnO_ = 2.9 V vs. NHE VB_TAPT-DMTP COF_ = 1.74 V vs. NHE	ZnO: by solvothermal method COF: interfacial Schiff base reaction	[89]
BiVO_4/_g-C_3_N_4_ (LHSCN)	S-scheme	CB_BiVO4_ = 0.46 V vs. NHE CB_LHSCN_ = −0.77 V vs. NHE VB_BiVO4_ = 2.63 V vs. NHE VB_LHSCN_ = 1.93 V vs. NHE	LHSCN: calcination method BiVO_4_: hydrothermal method	[90]
Decorated BiVO_4_ with Carbon Dots	e^−^ sink	CB_BiVO4_ = −0.01 V vs. NHE CB_composite_ = −0.35 V vs. NHE VB_BiVO4_ = 2.38 V vs. NHE VB_composite_ = 2.03 V vs. NHE	Carbon dots: hydrothermal method CQDs/BiVO_4_: hydrothermal method	[94]
Ti_3_C_2_/g-C_3_N_4_ (PGCN)	Schottky	CB_PGCN_ = −0.35 V vs. NHE VB_PGCN_ = 1.72 V vs. NHE	PGCN: Thermal polymerization of melamine Ti_3_C_2_ QDs: etching of Ti_3_AlC_2_ powder with HF	[96]
CQDs/FeP	e^−^ sink	CB_CDs/FeP_ = −0.088 V vs. RHE VB_CDs/Fe_ = 1.579 V vs. RHE	CDs: Electrochemical corrosion of graphite rod Carbon dots/FeP: hydrothermal method	[92]

**Table 3:** Photocatalytic performance of composites in contaminant degradation.

Heterostructures	System	Contaminant	Irradiation Conditions	Catalyst Loading *	Photocatalytic Performance **	Reusability (Cycles)	Ref.
Heptazine-COF@TiO_2_		100 mL Acetaminophen (10 ppm)	300 W Xe lamp (λ > 400 nm) 60 min	100 mg (1 g L^−1^)	99% (0.758 min^−1^, no ads after 60 min)	6	[102]
Ag-BiVO_4_@MXene		70 mL of Congo red (10 ppm) Crystal violet (5 ppm) Ciprofloxacin (5 ppm)	200 W Tungsten bulb 140 min	30 mg (0.43 g L^−1^)	Congo red: 72.9% (0.008 min^−1^) Crystal violet: 69.7% (0.008 min^−1^) Ciprofloxacin: 75.1% (0.009 min^−1^)		[103] ***
ZnO to MoS_2_		100 mL ciprofloxacin (0.05 mM)	250 W Metal halide lamp 120 min	50 mg (0.5 g L^−1^)	81%		[104]
g-C_3_N_4_/CQD onto a carbon cloth		40 mL ciprofloxacin (5 mg L^−1^)	250 W Xe lamp 60 min	Film	100% (minor ads after 15 min)	5	[105]
BiVO_4_@ g-C_3_N_5_	Z	20 mL ciprofloxacin (20 mg L^−1^)	Sunlight (842 W m^−2^) 120 min	20 mg (1 g L^−1^)	90.2% (0.015 min^−1^)	4	[106]
g-C_3_N_4_ with Ti_3_C_2_		50 mL cefixime (5 × 10^−5^ M)	Visible irradiation (2 × 45 W fluorescence lamps) 105 min	20 mg (0.4 g L^−1^)	64.69% (8.3% ads)		[107]
g-C_3_N_4_ with Ti_3_C_2_		clindamycin (125 mg L^−1^)	High-pressure lamp (100 mW cm^−2^) 120 min	10 mg L^−1^	92% (0.015 min^−1^, ~25% ads after 10 min) 38% mineralization	4	[108]
MoS_2/_BiVO_4_	p-n	100 mL tetracycline hydrochloride (5 mg L^−1^)	300 W Xe lamp (λ > 420 nm) 90 min	50 mg (0.5 g L^−1^)	90% removal (0.021 min^−1^, ~40% ads after 30 min)	3	[79]
COFs with BiVO_4_	II	100 mL tetracycline (20 mg L^−1^)	Xe lamp 90 min	20 mg (0.2 g L^−1^)	93.01% (0.067 min^−1^, ~30% ads after 30 min)	3	[71]
BiVO_4_/g-C_3_N_4_	S	50 mL tetracycline (40 mg L^−1^)	300 W Xe lamp (248 mW cm^−2^) 180 min	30 mg (0.6 g L^−1^)	~95% r (0.062 min^−1^, ~30% ads after 30 min)	5	[90]
g-C_3_N_4_/BiVO_4_	Z	100 mL RhB (20 mg L^−1^)	Xe lamp (350 W) for 5 h	0.5 g (5 g L^−1^)	90% (0.004 min^−1^, ~30% ads after 30 min)	4	[84]
g-C_3_N_4_/MoS_2_	Z	45 mL RhB (15 mg/L)	500 W Xe lamp (λ > 400 nm) for 2.5 h	20 mg (0.44 g L^−1^)	100% (0.040 min^−1^, ~15% ads after 30 min)		[85] ***
ZnO@Ti_3_C_2_	Schottky	20 mL MB (20 mg L^−1^)	300 W Xe lamp with UV filter 180 min	20 mg (1 g L^−1^)	~94.84% (0.012 min^−1^)	3	[95]
ZnO/V_2_C-MXene		10 mL MB (10^−5^ M)	Xe lamp (500 W)	50 mg (5 g L^−1^)	99.8% (0.054 min^−1^, 13% ads after 30 min)	4	[111]
QCDs/Ti_3_C_2_(OH)_2_		10 mL Malachite Green (75 ppm), pH 6	Visible light (Halogen lamp) 30 min	2 to 10 mg (max 1 g L^−1^)	Almost 100% (optimal conditions) (0.138 min^−1^)	3	[112]
PCDots-C_3_N_4_		50 mL Bisphenol A (10 mg/L) or 10 mL Tetracycline (10 mg L^−1^)	500 W Xe lamp (λ > 420 nm) 60 min	25 mg (0.5 g L^−1^)	TC: 76% (0.141 min^−1^) BPA: 100%		[62]
BiVO_4_ with CQDs		100 mL benzyl paraben (10 mg L^−1^)	300 W Xe lamp 150 min	0.1g (1 g L^−1^)	85.4%	4	[94]
BiVO_4_/α-Fe_2_O_3_		Benzene Degradation 100 mg m^−3^	8W UV lamp (365 nm, 1.52 mW cm^−2^) for 3.5 h	0.5 g	66.8% degradation (~25% ads after 30 min) 76.5% mineralization		[113]
Ti_3_C_2_-TiO_2_		10 ppm o-xylene (40 sccm)	300 W Xe lamp 180 min	100 mg into EtOH	77.3% reduction (mineralization released 37 ppm CO_2_)	4	[114]

* The reported catalyst dosage in g L^−1^ was estimated in the present review. ** Whenever possible, constant rate and adsorption percentages (ads) are given in parentheses. *** Incorporation of extra additives influenced the performance of the binary heterostructure.

**Table 4:** Antibacterial properties of photocatalytic composites.

Heterostructures	System	Contaminant	Irradiation Conditions	Catalyst Loading *	Photocatalytic Performance **	Reusability (Cycles)	Ref.
MoS_2_/N-CQDs (coating onto Ti sheet)		100 μL *S. aureus* (10^7^ cfu/mL) *E. coli* (10^7^ cfu/mL)	600 nm (0.35W cm^−2^) 15 min		Almost complete disinfection (minor inactivation in the dark)	Triplicate	[119]
MoS_2_/BiVO_4_	Z	*P. aeruginosa* (2 × 10^6^ cfu/mL) *S. aureus* (2 × 10^6^ cfu/mL)	500 W Xe lamp 90 min		Almost complete disinfection (less than 20% inactivation in the dark)	6	[66]
CoP/MoS_2_		1 mL saline solution of *S. aureus* (10^7^ cfu/mL)	75 W LEDs 15 min	1 mg (1 g L^−1^)	Almost complete disinfection (39.8% inactivation in the dark)	4	[120]
COF@CDs		*E. coli*	300 W Xe lamp (λ > 420 nm, 100 mW cm^−2^) Up to 60 min	50 mg L^−1^	Over 95% (8.3-fold decrease in survival rate compared to COF)		[121]

* The reported catalyst dosage in g L^−1^ was estimated in the present review. ** Whenever possible, constant rate and adsorption percentages (ads) are given in parentheses.

**Table 5:** Photocatalytic performance of composites in the reduction of Cr(VI).

Heterostructures	System	Contaminant	Irradiation Conditions	Catalyst Loading *	Photocatalytic Performance **	Reusability (Cycles)	Ref.
BiVO_4_@ g-C_3_N_5_	Z	20 mL Cr(VI) (5 mg L^−1^)	Sunlight (842 W m^−2^) 120 min	0.02 g L^−1^	91.4% (0.016 min^−1^)	4	[106]
C-QDs/TiO_2_ thin films		10 mL Cr(VI) (5 mg L^−1^) PNP (10 mg L^−1^) MB (5 mg L^−1^) or RhB (4 mg L^−1^)	UV-A fluorescent lamps (0.5 mW cm^−2^) 120 min	Film	56.0% Cr(VI) (0.006 min^−1^, 20% ads after 1 h) 87.5% PNP (0.017 min^−1^, 40% ads after 1 h) 81.5% MB (0.014 min^−1^, 30% ads after 1 h) 79.8% RhB (0.013 min^−1^, no ads after 1 h)	4	[125]
MoS_2_/α-Fe_2_O_3_		10 mL Tetracycline (20 ppm)	UV-A fluorescent lamps (0.23 mW cm^−2^) 40 min	0.2 g L^−1^	~ 70% TC (~25% ads after 1 h)		[126]
		50 mL Cr(VI) (5 ppm)		5 mg (0.1 g L^−1^)	Cr(VI) ~100% (~60% ads after 1 h)		
TPBPD-COF/g-C_3_N_4_	II	20 mL Cr(VI) (10 mg L^−1^), pH3)	300 W Xe lamp (λ > 400 nm) 120 min	20 mg (1 g L^−1^)	100% (0.141 min^−1^, 25% ads after 2 h)	4	[65]

* The reported catalyst dosage in g L^−1^ was estimated in the present review. ** Whenever possible, constant rate and adsorption percentages (ads) are given in parentheses.

**Table 6:** Photocatalytic performance of composites in H_2_ evolution.

Heterostructure	Junction	Irradiation Conditions	Catalyst Loading *	Products	Apparent Quantum Efficiency	Sacrificial Agent and Reusability	Ref.
g-C_3_N_4/_Ni_2_P	Z	500 W Xe lamp (100 mW cm^−2^, λ > 400 nm) 4 h	3 mg into 50 mL H_2_O (0.06 g L^−1^)	561 μmol g^−1^ h ^−1^ (100 times higher than g-C_3_N_4_)		TEOA (10% *v*/*v*, 50 mL) 4 cyclic experiments	[82]
Zn-Ni_2_P/g-C_3_N_4_	Z	300 W Xe lamp (λ > 420 nm) 1 h	50mg into 100 mL H_2_O (0.5 g L^−1^)	Ni_2_P/g-C_3_N_4_: 3758 μmol g^−1^ Zn-Ni_2_P/g-C_3_N_4_ (visible): 7539 μmol g^−1^ Zn-Ni_2_P/g-C_3_N_4_ (solar): 13,969 μmol g^−1^	8.9% under 420 nm	15% TEOA	[83]
Ti_3_C_2_/g-C_3_N_4_	Schottky	300 W Xe lamp 5 h	10 mg into 80 mL solution (0.125 g L^−1^)	4040.95 μmol/g/h (15.24 times more than CN, 3.53 times higher than PGCN)	11.8% at 420 nm	10% vol. triethanolamine	[96]
Ni_12_P_5_ onto TpPa-1-COF		300 W Xe lamp (λ > 400 nm) 6 h	10 mg into 100 mL DI H_2_O (0.1 g L^−1^)	3.16 mmol g^−1^ h ^−1^ for optimal 1:8 ratio (19 times higher than without Ni_12_P_5_)		100mg/L Ascorbic acid	[131]
CoP decorated CTF-COF	I	300 W Xe lamp (λ > 420 nm) 5 h	20 mg into 72 mL H_2_O (0.25 g L^−1^))	261.7 μmol g^−1^ h ^−1^	0.64% at 420 nm	8 mL TEOA 4 cyclic experiments	[70]
TAPB-Tp-COF with NH_2_-Ti_3_C_2_T_x_		300 W Xe lamp (λ > 400 nm) 4 h	5 mg with 3% wt. H_2_PtCl_6_ co-catalyst in 50 DI water (0.1 g L^−1^)	3.36 mmol g^−1^ h ^−1^ increased (26.67 times higher than COF)		0.44g ascorbic acid 4 cyclic experiments	[74] **
Ni_2_P/TiO_2_, TiO_2_/Ti_3_C_2_, Ni_2_P/TiO_2_/Ti_3_C_2_ titania composites	e-sink	35 W UV lamp (20 mW cm^−2^) 4 h	100 mg into 130 mL (0.77 g L^−1^)	Ni_2_P/TiO_2_/Ti_3_C_2_: 9425 ppm g^−1^ h^−1^ (×2.77 compared to Ni_2_P/TiO_2_ and ×4.81 to TiO_2_/Ti_3_C_2_)		5% *v*/*v* glycerol	[93]
CQDs/FeP		300 W Xe lamp (700 nm ≥ λ ≥ 400 nm) 36 h	5 mg in 20 mL ultrapure water (0.25 g L^−1^)	33.85 μmol/h/g compared to 6.65 for FeP	0.703%_o_ at 500 nm		[92]
MoS_2_/Ti_3_C_2_		300 W Xe lamp (λ > 400 nm) 5 h	30 mg into 50 mL H_2_O (0.6 g g L^−1^) g L^−1^)	MoS_2_: 2626 μmol g^−1^ h^−1^ MoS_2_/Ti_3_C_2_: 6144 μmol g^−1^ h^−1^		methanol 30% 4 cyclic experiments	[132]
MoS_2_ @TPPy-COF	II	400 W Hg lamp 10 h	50 mg in 15 mL H_2_O (3.3 g L^−1^)	12,874 μmol g^−1^		10% *v*/*v* triethanolamine (TEOA) as a hole scavenger agent	[73]
MoS_2_/ZnO		300 W Xe lamp 5 h	50 mg into 100 mL Di H_2_O (0.5 g L^−1^)	235 μmol/g/h		0.2M Na_2_SO_3_	[104]

* The reported catalyst dosage in g L^−1^ was estimated in the present review. ** Incorporation of extra additives influenced the overall performance of the binary heterostructure.

**Table 7:** Performance of binary composites in photocatalytic reduction of CO_2_.

Heterostructure	Junction	Irradiation Conditions	Catalyst Loading *	Products	Comments	Ref.
MoS_2_ @TPPy-COF	II	445 nm white LED bulb 3 h	15 mg 10 mL of 1:1 ACN:H_2_O (1.5 g L^−1^)	HCOOH rate is 966 μmol g^−1^ in 3 h	1 mL triethanolamine (TEOA) as substitutional electron source	[73]
MoS_2_@COF	-	300 W Xe lamp (λ > 420 nm, 209 mW cm^−2^)	1 mg into 3 mL acetonitrile/1 mL H_2_O/1 mL triethanolamine (0.2 g L^−1^)	CO, CH_3_OH, CH_4_, C_2_H_6_ production rate up to 56.2 μmol⋅g^−1^1⋅h^−1^ with 83.8% of selectivity	[Ru(bpy)3]Cl_2_⋅6H_2_O as photosensitizer triethanolamine (TEOA) as hole-scavenger In situ DRIFTS	[134] *
IL@MTPA/BiVO_4_	Z	300 W Xe lamp 4 h	20 mg in 10 mL DI water (2 g L^−1^)	MTPA/BiVO_4_ 151 g^−1^ h^−1^ IL@MTPA/BiVO_4_ 903.6 μmol g^−1^ h^−1^, with nearly 100% selectivity to CO and CH_4_	6-fold enhancement compared to the non-confined composite and a 38-fold enhancement compared to the BiVO_4_ catalyst	[81] **
N-ZnO/TAPT-DMTP COF	S	300 W Xe lamp (1850 μW cm^−2^) 5 h	20 mg in 20 mL isopropyl alcohol (1 g L^−1^)	11.07 μmol g^−1^ of CO and 4.86 μmol g^−1^ of CH_4_ after		[89]
CDs modified 0D-ZnO	-	400 W Xe lamp 8 h	Gas flow reactor	CO evolution: 119 μmol g^−1^ h^−1^, surpassing the 32.2 μmol g^−1^ h^−1^ of pristine 0D-ZnO and 5.3 μmol g^−1^ h^−1^ of Degussa TiO_2_	In situ DRIFTS	[135]

* The reported catalyst dosage in g L^−1^ was estimated in the present review. ** Incorporation of extra additives influenced the overall performance of the binary heterostructure.

## Data Availability

No new data were created or analyzed in this study. Data sharing is not applicable to this article.

## Abbreviations

The following abbreviations are used in this manuscript:

SC	Semiconductor
E_g_	Energy band gap
e^−^	Photogenerated electron
h^+^	Photogenerated hole
CB	Conduction band
VB	Valence band
ROS	Reactive oxygen species
V_ox_	oxidation potential
V_red_	Reduction Potential
CDs	Carbon Dots
COFs	Covalent Organic Frameworks
TMPs	Transition metal phosphides
EPR	Electron Paramagnetic Resonance
DFT	Density Functional Theory
PL	Photoluminescence
DRIFTS	Diffuse Reflectance Infrared Fourier Transform Spectroscopy
ads	adsorption
